# Autologous blood injection intracoronary artery for treating slow‐flow and no‐reflow in acute coronary syndrome related to primary pci

**DOI:** 10.1002/ccr3.5328

**Published:** 2022-02-23

**Authors:** Lam Truong Hoai, Duy Nguyen Xuan, Hung Nguyen Duc, Long Nguyen Tuan

**Affiliations:** ^1^ Tam Anh Hospital Hanoi Capital Vietnam

**Keywords:** acute coronary syndrome, acute crisis thrombosis, autologous blood injection, balloon angioplasty, Slow‐flow and no‐reflow

## Abstract

Slow‐flow and no‐reflow phenomenon are taken to sudden loss of coronary artery flow, typically after stenting or angioplasty in primary PCI. Otherwise conventional therapy, we report a technique, which autologous blood into intracoronary to supply oxygen and break process thrombosis results in successfully management no‐reflow in primary PCI in ACS.

## Graphical abstract

Management of the slow‐flow and no‐reflow when the conventional therapy is not already. That may be a new technique that considers when hemodynamic unstable and is not suitable with the medication especially without IIb/IIIa inhibitor.

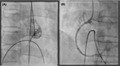

## 1 INTRODUCTION

Early revascularization of an intact‐related artery (IRA) plays an important role in ACS to prevent myocardium injury. With all the recent advances in equipment and techniques, revascularization has become a faster and good result. However, the no‐reflow phenomenon occurs in a considerable number of patients undergoing primary PCI ranging between 12% and 32.8%.^1^ This phenomenon is associated with arrhythmias, poor in‐hospital survival, and poor 1 year survival. Intracoronary vasodilators such as verapamil, nitroprusside, or adenosine are being administered for the treatment of no‐reflow via the microcatheter. But sometimes distal flow restoration is not satisfactory especially in patients with TIMI 0 flow because of hemodynamic unstable and heart shock result in less effective medication lead to the circuit pathology is that no flow, thrombosis, heart shock, arrhythmias with consequence death. The use of blood with high O_2_ saturation pump into the intracoronary to supply oxygen for the myocardium while the no‐reflow phenomenon occurs, and this helps to maintain flow in the vessel to prevent thrombosis. We report a clinical case using the self blood pump into the coronary artery to treat no flow intracoronary in ACS patients with primary PCI successfully.

## 2 PRESENTATION

A 61‐year‐old man with a history of hypertension was admitted to the hospital because of severe chest pain. In the emergency department, the patient suddenly cardiac arrest, and chest compressions immediately with Adrenaline 1 mg/1ml × 5 ampoules, after that monitoring showed V‐tach and defibrillation with 200 J. ECG showed BAV III, ST elevated in DII, DIII, aVF, and heart rate was 40 bpm. Blood pressure was 90/60 mmHg with Noreadrenalin 0.1 mcg/kg/h, Dobutamine 10 mcg/kg/h, and mechanical ventilation. Patient transferred to cath laboratory immediately with totally medication which was, Lidocaine 40 mg/2ml × 2 ampoules, Magnesisulfat 15% × 10ml × 2 ampoules, Lovenox 0,5 mg/kg, Aspirin × 300 mg, Ticagrelor 180 mg, and Rosuvastatin 40 mg.

**FIGURE 1:**
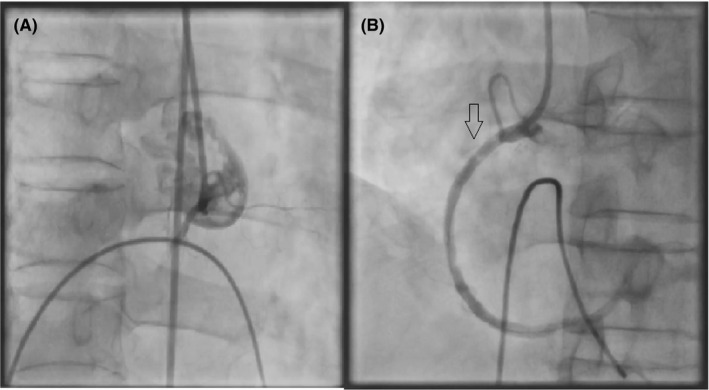
Angiography showed proximal RCA occlusion (A), the thrombus appearance at proximal RCA (B) (arrow)

A temporary pacemaker was implanted on the right femoral vein with HR 80 bpm, output 3 mv, sensing 3 mv. The angiography on the right femoral artery showed proximal RCA occlusion (Figure 1A). The thrombus aspiration device had been used, which was not effective. Balloon angioplasty which complaint ballon 2.5 × 20mm was used, angiogram showed RCA TIMI III flow and lesion were RCA I‐II and RCA III with 95% and 90% irrespectively. Stents were implanted with DES 3.5 × 40 mm (20 atm) for RCA I‐II, and DES 2.75 × 40 mm (12 atm) for RCA III‐PLV. The hemodynamic was stable BP 100/60 mmHg (with noradrenaline and dobutamine), and the TIMI III flow was shown on the angiogram. After 5 min, the angiogram showed thrombus in the proximal RCA (Figure 1B) (ACT was 230 s). With the mud thrombosis which the thrombus aspiration device was not effective, we decided active balloon angioplasty with a compliant balloon (3.5 × 20mm) to maintain the flow but as a result slow‐flow on the angiogram (TIMI I), which may be microvascular constriction result in hemodynamic unstable and arrhythmia (V‐tach or Vfib). The medication was indicated which was not suitable because of low BP and arrhythmia, including Adenosine, Nitroglycerin. To maintain flow in RCA, we decided active angioplasty balloon and the use of blood with high O_2_saturation pump into the intracoronary to supply oxygen for the myocardium while the no‐reflow phenomenon occurs through a contralateral femoral artery. Using syringe 10 ml pumped through thrombus aspiration device, hand force just enough to reduce erythrocyte rupture, with a frequency of about 5 pumps/min, active angioplasty balloon every 5 min to maintain open vessel lumen and maintain flow. Thrombus aspiration still measures coronary perfusion pressure which demonstrates perfusion in the coronary artery while could not measure pressure through guiding catheter. The patient became more stable, which arrhythmia and pressure drop were not apparent while we did the procedure.

After 1 h of active balloon angioplasty and pump blood with high O_2_ saturation, the angiogram showed TIMI III flow and thrombus apparent, hemodynamically stable, the patient was transferred to CCU for monitoring and treatment. After 3 days of wearing off ventilators and vasoconstriction. After a total of 10 days, the patient was discharged with a stable condition.

## 3 DISCUSSION

Originally, it was thought prolonged ischemia and extensive myocardial damage led to microvascular (capillary bed) damage, or microvascular vasoconstriction resulting in incomplete reperfusion, other factors have been thought to play an important role in the development of no‐reflow, Platelets may be implicated in slow‐flow and no‐reflow through several mechanisms, including microvascular vasoconstriction or obstruction by platelet aggregates. Although the exact mechanism of no‐reflow remains unknown, it is most likely complex and multi‐factorial. The selective use of glycoprotein IIb/IIIa inhibitors and thrombectomy devices during the intervention may be also appropriate in the selected case, but the mud thrombus almost failed with the thrombectomy device, and glycoprotein IIb/IIIa lack of randomized controlled clinical trials remains a limitation. Unfortunately, In Vietnam, the glycoprotein IIb/IIIa has not already. However, no‐reflow can still occur even under the best‐provided care, which emphasizes the need for more specific mechanical and/or medical treatments. This is particularly true in the setting of an acute STEMI. Various vasodilators have been shown to affect some cases of success including nicardipine, nitroprusside, and verapamil, but the longer‐acting drugs are somewhat limited by significant hemodynamic unstable and arrhythmia, negative inotropy, which are of particular importance in the acutely ischemic heart. Two drugs in particular (nitroprusside and adenosine) have been studied as possible adjuncts to reperfusion therapy in some trials^2^, ^3^ but the effect is not strong enough to succeed in all cases especially in an unstable hemodynamic and significantly arrhythmias situation while continuously autologous blood injection into the intracoronary artery through aspiration thrombus device is an important role to good choice to keep flowing compensate flow disturbance.

Autologous blood injection which is high O_2_ saturation into the coronary artery with the pressure and velocity stable to demand myocardium to reduce the myocardium injury when slow‐flow and no‐reflow occurred, which is promised technique. In addition, active balloon angioplasty to break thrombus formation and fibrin results in decreased crisis thrombosis and maintain flow. As a result, the coronary flow is preserved. Keep doing it until the crisis thrombosis pass away and the flow is improved. The time to pass to crisis thrombosis is important due to such thrombin activity and fibrin polymerization fronts typically travel slowly at a rate of 3 mm in 60 min, consistent with simulation.^4^ Slow traveling wave propagation to the physics in stagnant blood clotting, while blood injection can warrant the pressure and velocity of flow with high oxygen while the patient suffers from cardiac shock, is that the medication show less effect. Moreover, in a critical setting, continuous autologous blood injection intracoronary artery can play an important role until all disturbances disappeared. Similar to ECMO and IABP are ensure hemodynamic and oxygen for myocardium by systemic support, and autologous blood injection to supply for local ischemic myocardium.

Virchow's triad shows the 3rd factor is that vessel injury, stasis of blood, and hypercoagulability of blood, which is correlative with the mechanism of crisis thrombosis after stenting due to STEMI, while blood artery can cover include stasis of blood by injecting blood into intracoronary with stable pressure and active balloon angioplasty to maintain flow, to supply oxygen for myocardium to reduce endothelium damages, necrosis, supplying more fibrinogen form another site where the crisis thrombosis does not occur. Moreover, slow‐flow and no‐reflow could preserve. This is the reason why we should keep doing procedures while waiting for the flow preserve to become normal.

## 4 CONCLUSION

Autologous blood which has high oxygen injected to the intracoronary artery and active balloon angioplasty is a role of treating slow‐flow and no‐reflow when the other conventional therapy is not suitable especially in hemodynamic unstable and arrhythmias situations. Keep flowing and demand high blood oxygen to reduce endothelial damage and myocardium necrosis result in increase survival when crisis thrombosis and disturbance flow appearance.

## CONFLICTS OF INTEREST

Conflict of interest relevant to this article was not reported.

## AUTHOR CONTRIBUTIONS

All authors contributed to data analysis, drafting, or revising the article, have agreed on the journal to which the article will be submitted, gave final approval of the version to be published, and agree to be accountable for all aspects of the work.

## CONSENT

Written informed consent form was given to patient.

## STATEMENT INDICATING

“Written informed consent was obtained from the patient to publish this report in accordance with the journal's patient consent policy.”

## Data Availability

Availability of data and materials supporting our findings will be shared upon request.
